# The Association of Demographic, Socioeconomic, and Geographic Factors with Potentially Preventable Emergency Department Utilization

**DOI:** 10.5811/westjem.2021.5.50233

**Published:** 2021-10-27

**Authors:** Lucas C. Carlson, Kori S. Zachrison, Brian J. Yun, Gia Ciccolo, Benjamin A. White, Carlos A. Camargo, Margaret E. Samuels-Kalow

**Affiliations:** * Partners HealthCare, Population Health Management, Somerville, Massachusetts; † Brigham and Women’s Hospital, Department of Emergency Medicine, Boston, Massachusetts; ‡ Massachusetts General Hospital, Department of Emergency Medicine, Boston, Massachusetts

## Abstract

**Introduction:**

Prevention quality indicators (PQI) are a set of measures used to characterize healthcare utilization for conditions identified as being potentially preventable with high quality ambulatory care. These indicators have recently been adapted for emergency department (ED) patient presentations. In this study the authors sought to identify opportunities to potentially prevent emergency conditions and to strengthen systems of ambulatory care by analyzing patterns of ED utilization for PQI conditions.

**Methods:**

Using multivariable logistic regression, the authors analyzed the relationship of patient demographics and neighborhood-level socioeconomic indicators with ED utilization for PQI conditions based on ED visits at an urban, academic medical center in 2017. We also used multilevel modeling to assess the contribution of these variables to neighborhood-level variation in the likelihood of an ED visit for a PQI condition.

**Results:**

Of the included 98,522 visits, 17.5% were categorized as potentially preventable based on the ED PQI definition. On multivariate analysis, age < 18 years, Black race, and Medicare insurance had the strongest positive associations with PQI visits, with adjusted odds ratios (aOR) of 1.41 (95% confidence interval [CI], 1.29, 1.56), 1.40 (95% CI, 1.22, 1.61), and 1.40 (95% CI, 1.28, 1.54), respectively. All included neighborhood-level socioeconomic variables were significantly associated with PQI visit likelihood on univariable analysis; however; only level of education attainment and private car ownership remained significantly associated in the multivariable model, with aOR of 1.13 (95% CI, 1.10, 1.17) and 0.96 (95% CI, 0.93, 0.99) per quartile increase, respectively. This multilevel model demonstrated significant variation in PQI visit likelihood attributable to neighborhood, with interclass correlation decreasing from 5.92% (95% CI, 5.20, 6.73) in our unadjusted model to 4.12% (95% CI, 3.47, 4.87) in our fully adjusted model and median OR similarly decreasing from 1.54 to 1.43.

**Conclusion:**

Demographic and local socioeconomic factors were significantly associated with ED utilization for PQI conditions. Future public health efforts can bolster efforts to target underlying social drivers of health and support access to primary care for patients who are Black, Latino, pediatric, or Medicare-dependent to potentially prevent emergency conditions (and the need for emergency care). Further research is needed to explore other factors beyond demographics and socioeconomic characteristics driving spatial variation in ED PQI visit likelihood.

## INTRODUCTION

Emergency conditions are defined by the manifestation of acute symptoms that may represent a threat to life, limb, or an individual’s future health, and accordingly require urgent evaluation and potential intervention.^1^ While a number of studies have attempted to retrospectively infer the need for emergency care, and which emergency visits were “unnecessary” or “avoidable,” based on diagnoses obtained after a patient’s evaluation in the emergency department (ED),^2^–^4^ increasingly evidence has suggested that the need for emergency care can only be reliably determined by the patient experiencing symptoms at the time of presentation.^5^,^6^ Still, like other health conditions some emergency conditions and the need for emergency care can be prevented, both through primary prevention efforts such as influenza vaccinations, as well as secondary prevention such as coronary artery disease maintenance in primary care.^7^,^8^ Correspondingly, acute outpatient visits and the use of alternative sites of care such as urgent care centers, when available, accessible, and appropriate, could also be seen as a kind of tertiary prevention.^9^

To advance our understanding of potentially preventable acute care utilization, recent efforts have sought to define, measure, and characterize utilization for acute conditions that could have been prevented with robust primary care.^10^ The Prevention Quality Indicators (PQI) are a set of measures defined by the Agency for Healthcare Research and Quality based on rates of hospitalization for a pre-specified list of conditions identified as ambulatory care sensitive conditions (ACSC), or conditions for which hospitalization could have been prevented with high quality ambulatory care. These measures have been used to identify opportunities to improve and strengthen primary and preventive care.^11^ The PQIs have recently been adapted to ED presentations using a similar list of ED diagnoses, termed the ED PQIs, which can also be used to identify areas or populations for which strengthened ambulatory care systems could potentially prevent the need for emergency care.^12^

The authors investigated ED PQIs to measure and characterize potentially preventable ED utilization at a large, urban, academic medical center. Specifically, he analyzed the relationship of demographics and neighborhood-level socioeconomic indicators with potentially preventable ED utilization. He also explored the degree to which an individual’s neighborhood characteristics contribute to preventable ED utilization.

## METHODS

### Data Sources

In this study, the authors used clinical data from ED visits to a large, urban, academic medical center, which sees approximately 110,000 ED visits per year, to analyze the relationship of demographics, neighborhood-level socioeconomic indicators, and potentially preventable ED utilization. All patient visits to the ED during calendar year 2017 were included in the analysis, including visits to the pediatric section of the ED. This study was reviewed and approved by the local institutional review board.

**Population Health Research Capsule**

What do we already know about this issue?

*Patterns of emergency department (ED) utilization can be used to identify opportunities to potentially prevent emergency conditions and to strengthen systems of ambulatory care.*

What was the research question?

*How do demographic, neighborhood, and socioeconomic indicators relate to ED utilization for preventable conditions?*

What was the major finding of the study?

*Race, age, socioeconomic variables, and neighborhood were significantly associated with ED use for preventable conditions.*

How does this improve population health?

*Future efforts can potentially prevent emergency conditions and the need for emergency care by targeting the underlying patient and socioeconomic factors driving ED use.*

The authors obtained demographic information (age, gender, race/ethnicity), home addresses, and clinical data (ED diagnosis codes, ED disposition) from the electronic health records for all included ED visits. As the primary outcome of interest, he used the ED PQIs defined by Davies et al,^12^ converted from the *International Classification of Diseases, 9th Revision* (ICD-9) to ICD-10 based on the National Bureau of Economic Research crosswalk,^13^ to classify ED visits as non-PQI visits or PQI visits (ie, those for which high quality ambulatory care could have potentially prevented the need for emergency care). The authors specifically used PQI numerator definitions to categorize PQI status of included visits. These include dental condition, chronic ACSC (eg, heart failure, chronic kidney disease), acute ACSC (eg, acute otitis media, cellulitis), asthma, and back pain – each with specific associated ICD codes, inclusion criteria, and exclusion criteria. Please see Appendix 1 for more information regarding ED PQI conditions (ED PQI ICD-10 codes available from authors upon request). The ED visits were then geocoded based on patient home address and imported to ArcGIS 10.1 (Environmental Systems Research Institute; Redlands, CA) for geospatial analysis.

After projecting addresses in North American Datum 1983 (NAD83) Massachusetts (MA) state plane coordinate system, the authors tagged ED visits to the patient’s respective Census Block Group (CBG) and calculated each address’ Euclidean (straight-line) distance to the hospital. The CBG was chosen as the unit of inclusion given that it is the smallest geographic unit available with the corresponding census data, which in general encompass a population of between 600–3000 people, and has been used for previous area-level health analyses.^15^ Prior healthcare utilization research has demonstrated Euclidean distance to closely correspond with travel times.^14^ Seven CBG-level socioeconomic indicators were selected for inclusion in the analysis based on prior studies of healthcare utilization and socioeconomic disadvantage, specifically the following: percent of adults without a high school diploma; percent of households with a single parent; percent of households receiving public assistance; percent of households without a private car; percent of families with income under 100% of the federal poverty line (FPL); percent of families with income under 200% of the FPL; and unemployment rates.^15^ The CBG-level values were obtained for each variable from the 2013–2017 American Community Survey 5-year estimates.^16^

### Data Analysis

Geospatial, demographic, and corresponding clinical data were then imported to STATA 13.1 (Statacorp LP, College Station, TX) for further statistical analysis. One-way comparisons between PQI and non-PQI visits were performed for demographic and neighborhood-level variables using Student’s t-tests. The authors used multivariable logistic regression to calculate adjusted odds ratios (aOR) of the likelihood of the ED visit being for a PQI using demographic, visit, and CBG-level socioeconomic covariates, with error clustered at the CBG-level. These covariates were selected a priori based on prior research demonstrating the importance of these variables in predicting healthcare utilization.^15^ For the logistic regression, CBG-level socioeconomic variables were converted from percentages to CBG quartiles for ease of interpretability. Collinearity between the included CBG-level socioeconomic variables were tested using tolerance and variance inflation factor, and there was no evidence of severe collinearity that may have significantly impacted the findings.

The authors also used multilevel modeling to assess the contribution of these variables to the likelihood of a visit being for a PQI condition. A series of hierarchal logistic regression models were performed with patient-level and CBG-level variables. The auhor first developed an empty model (Model 0), adjusting for neighborhood-level variation with random intercepts. Model 1 added patient-level demographic and visit characteristics to the model 0. Model 2 added hospital distance to Model 1. The final, full model (Model 3) added neighborhood socioeconomic variables to Model 2. For each model, intraclass correlation coefficients (ICC) and median odds ratios (MOR) were calculated to characterize the degree to which each group of variables contributed to PQI visit likelihood.^17^

## RESULTS

**Figure 1:**
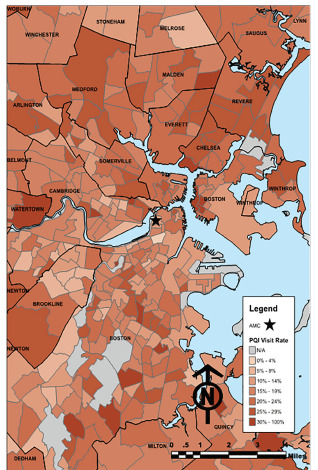
Percent of emergency department visits for prevention quality indicator conditions by census tract. *AMC*, academic medical center; *PQI*, prevention quality indicator; *N/A*: census tract with <10 visits total.

A total of 108,872 ED visits during 2017 were available for inclusion in the study. Of these, 108,069 (99.3%) were successfully geocoded, and 98,522 (91.2%) were both located in MA and had complete CBG data available from the US Census data bank (Figure 1). According to the ED PQI definition, 17,204 (17.5%) of these 98,522 visits were for PQIs. The PQI and non-PQI visits differed significantly by age, gender, race/ethnicity, insurance status, hospital distance, and each of the tested CBG-level socioeconomic indicators (Table 1). In general, patients with PQI ED visits were older, less likely to have private insurance, and were from neighborhoods with somewhat higher measures of socioeconomic disadvantage.

In the logistic regression analysis, patient age, race/ethnicity, insurance status, season, percent of adults without a high school diploma and percent of households without a private car were significantly associated with likelihood that an ED visit was for a PQI condition (Table 2). Neighborhood-level rates of adults without a high school diploma, by quartile, had an aOR of 1.13 (95% confidence interval [CI], 1.10, 1.17). Percent of households without a private car, by neighborhood quartile, was negatively associated with PQI visit likelihood, with an aOR of 0.96 (95% CI, 0.93, 0.99). After adjusting for other included variables, other neighborhood factors (percent of households with a single parent; percent of households receiving public assistance; percent of families with income under 100% of the federal poverty line (FPL); percent of families with income under 200% of the FPL; and unemployment rates) were not significantly associated with likelihood of having visited the ED for a PQI condition.

In our multilevel model, the intraclass correlation coefficient (ICC) for the unadjusted model was 5.92% (95% CI, 5.20, 6.73), indicating variation in PQI visit likelihood attributable to patient neighborhood (Table 3). The MOR in the unadjusted model was 1.54, also indicating that CBG was associated with PQI visit likelihood relative to other tested variables.^17^ After adjusting for patient demographic factors, the ICC decreased to 4.97% (95% CI, 4.28, 5.80); and in the fully adjusted model, including neighborhood-level socioeconomic indicators, the ICC was lower than the unadjusted model, 4.12% (95% CI, 3.47, 4.87), and the MOR was lower as well: 1.43. These findings support that the included neighborhood-level socioeconomic variables explained some of the variation attributable to patient neighborhood, but that there was still significant residual spatial variation unexplained by these factors.

## DISCUSSION

Using ED PQI definitions, the authors found that demographic and neighborhood factors are significantly associated with ED utilization for ACSCs. This study adds to the existing literature regarding ED utilization patterns and socioeconomic drivers of health by characterizing preventable ED utilization using the ED PQI definitions, as well as applying the ED PQIs using updated ICD-10 definitions.^12^,^15^,^18^,^19^ Overall, patient age, race, and insurance had the strongest relationships with ED PQI visit likelihood. Patient age <18 years was associated with more than 40% higher odds of visiting the ED for an ED PQI condition. This is likely due to varying patterns of ED utilization between pediatric and non-pediatric patient populations or differing thresholds for parents/guardians to decide to visit an ED with a pediatric patient for conditions otherwise considered amenable to primary care. This may also be due in part to confounding by varying incidences of ED PQI conditions between pediatric and non-pediatric age groups as well as age specifications used within the ED PQI numerator definitions.

Uninsured status was also strongly associated with decreased likelihood of using the ED for an ED PQI condition. This may suggest that patients without insurance and therefore unshielded from healthcare costs are less likely to use the ED for ED PQI conditions; however, this finding may also be due to confounding as individuals who are healthier and with fewer chronic conditions may be less likely to seek or obtain insurance.^28^ It is also unclear, however, whether this is generalizable outside of Massachusetts, where the uninsured rate is only 3% compared with >9% nationwide.^29^

Although each of the tested socioeconomic variables was associated with PQI visit likelihood on univariable analysis, after adjusting for demographic and other neighborhood-level socioeconomic indicators, only percent of adults without a high school diploma significantly predicted higher PQI visit likelihood, with every increase in quartile being associated with a 13% increase in the odds of the ED visit being for a PQI condition.

The strong relationship between preventable utilization and level of educational attainment has been noted in other settings and is likely multifactorial.^20^ In regard to preventable emergency conditions, this finding may indicate that barriers to ambulatory primary care mirror barriers to other public services such as education. This relationship may also be tied to health literacy and numeracy, in that many aspects involved in coordinating an individual’s care rely on these proficiencies.^21^ Lastly, it may also be related to constraints around the particular types of jobs available to individuals who do not have a high school diploma, as certain jobs may be more flexible in allowing an individual to coordinate outpatient care during standard business hours, and accordingly be less reliant on after-hours emergency care. Further research will be necessary to better understand the factors underpinning this association.

Interestingly, although PQI visits were greater for patients from areas with lower percentages of households without a private car, after adjusting for demographics and other socioeconomic variables, percent of households without a private car was negatively associated with likelihood of PQI visit.^22^ This finding is somewhat counterintuitive and contrary to prior studies of ED utilization,^22^ but it suggests that there are other factors related to higher vehicle ownership rates that, once disentangled from other socioeconomic factors, may lead to increases in preventable ED utilization. This may be related to the robust public transportation system available in Boston and to the fact that a large percentage of individuals living in Boston do not own private cars.^23^ However, this finding may also be a function of patients’ access to emergency care, underlying disproportionate burden of other health conditions in this population, or a different threshold to seek emergency care for those with ready access to private transportation.

According to the authors’ analysis, PQI ED visits were more common among racial/ethnic minorities and patients from neighborhoods with higher levels of socioeconomic disadvantage. This presumably reflects existing inequity in access to primary care and greater overall risk likely related to socioeconomic drivers, thus indicating a need for strengthened systems of care for these populations.^24^ The authors also found that PQI visit rates were significantly higher among patients with Medicare. This finding suggests that there is substantial opportunity to improve ambulatory care and chronic disease management among the Medicare population in this setting, and accordingly decrease their need for emergency care. In this analysis, the authors also found that there was no significant difference in PQI ED visit likelihood between Medicaid- and commercially insured patient populations, supporting prior research challenging assertions that patients with Medicaid more frequently use the ED for non-urgent or routine care.^25^

Although PQI visit likelihood was significantly associated with both patient demographic and neighborhood socioeconomic variables, there was still significant residual variation at the neighborhood-level unexplained by these factors. This finding could be related to the organization of public and private transportation systems, local hospital preferences and care-seeking patterns, or neighborhood-level variation in social risk unaccounted for by the included socioeconomic variables. These findings indicate that although socioeconomic factors are important drivers of preventable ED utilization, there are still other factors linked to place of residence that affect patterns of emergency care utilization. These may include neighborhood access to other providers of acute unscheduled care (eg, urgent care centers); local practices among primary care providers with regard to ED referral; and financial frameworks/incentives of area healthcare systems. These factors can be further explored in future geospatial analyses. However, regardless of the factors underlying this association, this study demonstrates the importance of place for patients’ health status and needs. The public health community can further use this knowledge to geographically target prevention efforts and programs aimed at supporting access to primary care and other interventions to address social determinants of health.

In addition, the finding that patients from areas with higher measures of socioeconomic stress were more likely to visit the ED for conditions that may otherwise be considered preventable by robust, reliable primary care further supports the position of the ED as a critical element of the healthcare safety net.^30^,^31^ The fact that patients from disadvantaged areas are more likely to rely on the ED for routine care, or even at times preventative care, only further reinforces the need for robust emergency care systems as an essential part of the fabric of the public health system.

## LIMITATIONS

This study has several potential limitations. First, it is an analysis of the experience of a single ED, and therefore these findings may not necessarily be generalizable to other EDs and healthcare systems outside of this specific context. Similarly, Boston is in a unique healthcare market with broad engagement in accountable care organizations and low rates of uninsured patients, which may differ substantially from other settings.^26^,^27^ Next, the authors oincluded visits only by patients with home addresses that were able to be successfully geocoded, consequently excluding undomiciled patients from our analysis; thus, these results do not reflect the likely substantial impact of socioeconomic drivers on utilization among this population. Neither did the data include time of day or day of the week of the ED visit, therefore making it impossible to comment on how these factors may have affected ED utilization for PQI conditions. Also, although it has been shown to be reliable in previous health services research, Euclidean distance was relied upon for distance calculations.^14^

In addition, although the ED PQI definitions were developed in a robust fashion, the preventability of these conditions is not definitive but rather exists on a spectrum. For example, an older adult with an upper respiratory infection presenting as shortness of breath may require further evaluation to rule out congestive heart failure or pulmonary embolism based on their presentation, and therefore cannot be characterized as preventable. Future assessments of ED PQI definitions could aim to evaluate the correlation between chief complaints and ED PQI diagnoses to further explore this question. Furthermore, many of the ED visits that could have been prevented with ambulatory care are not necessarily categorized as ACSCs using the PQI definition. Lastly, PQIs are defined as measures based on rates of utilization for an area or populations, including specific denominators of utilization. In this analysis, however, as the authors was analyzing visit-level data, he used only the definitions for the PQI numerators.

## CONCLUSION

This analysis provides new data and a more nuanced understanding of patterns of ED utilization for ambulatory care sensitive conditions and opportunities for the prevention of emergency conditions. According to these findings, demographic and socioeconomic variables both in part explain neighborhood-level variation in ED utilization for PQI conditions. Future efforts to prevent emergency conditions and the need for emergency care can aim to do so by targeting efforts to pediatric, Black, Latino, and Medicare patient populations, as well as targeting the underlying socioeconomic factors driving utilization. Further research is also needed to explore other potentially modifiable factors beyond patient demographics and socioeconomic characteristics driving spatial variation in ED Patient Quality Indicators visit likelihood.

## Supplementary Information



## Figures and Tables

**Table 1:** Comparison of demographic and neighborhood-level socioeconomic variables among Prevention Quality Indicators (PQI) and non-PQI visits.

Variable	Non-PQI	PQI	p-value
Mean age (years)	43.2	56.2	<0.0001
Age <18 (%)	12.3	6.4	<0.0001
Age ≥65 (%)	20.0	40.9	<0.0001
Female (%)	47.3	48.5	0.003
Race/ethnicity (%)			
White	63.1	65.9	<0.0001
Black	10.1	11.8	<0.0001
Latino	15.5	13.9	<0.0001
Asian	4.4	3.3	<0.0001
Other	10.3	8.2	<0.0001
Primary insurance (%)			
Medicaid	15.5	13.0	<0.0001
Medicare	20.6	40.8	<0.0001
Private	59.4	43.4	<0.0001
Uninsured	4.5	2.7	<0.0001
Hospital distance (miles)	9.9	9.1	<0.0001
CBG characteristic (%)			
People >25 years without HS diploma	12.7	14.1	<0.0001
Households with single parent	28.6	29.9	<0.0001
Households receiving public assistance	3.0	3.1	0.002
Households without private car	22.6	22.9	0.04
Families with income <100% FPL	10.2	10.9	<0.0001
Families with income <200% FPL	29.1	30.2	<0.0001
Adults that are unemployed	6.5	6.7	0.01

*PQI*, Prevention Quality Indicator; *CBG*, Census Block Group; *HS*, high school; *FPL*, federal poverty line.

**Table 2:** Multivariable logistic regression results of the likelihood of an ED visit being categorized as a Prevention Quality Indicator (PQI) vs non-PQI visit.

Variable	Odds ratio (95% CI)
Age (increasing by decile)	1.02 (1.02 – 1.02)
Pediatric visit (age <18 years)	1.41 (1.29 – 1.56)
Female	1.01 (0.96 – 1.06)
Race/ethnicity (relative to Asian)	
White	1.05 (0.94 – 1.18)
Black	1.40 (1.22 – 1.61)
Latino	1.22 (1.08 – 1.38)
Other	1.00 (0.88 – 1.14)
Insurance (relative to Medicaid)	
Medicare	1.40 (1.28 – 1.54)
Private	0.94 (0.87 – 1.01)
Uninsured	0.79 (0.69 – 0.90)
Hospital distance (miles)	0.99 (0.99 – 1.00)
Season (relative to Fall)	
Spring	1.03 (0.98 – 1.08)
Summer	0.88 (0.84 – 0.93)
Winter	1.10 (1.05 – 1.16)
CBG characteristic (quartile)	
People >25 years without HS diploma	1.13 (1.10 – 1.17)
Households with single parent	1.03 (1.00 – 1.07)
Households receiving public assistance	1.00 (0.97 – 1.03)
Households without private car	0.96 (0.93 – 0.99)
Families with income <100% FPL	1.01 (0.97 – 1.05)
Families with income <200% FPL	1.01 (0.96 – 1.06)
Adults that are unemployed	1.00 (0.98 – 1.03)

*CI*, confidence interval; *CBG*, Census Block Group; *HS*, high school; *FPL*, federal poverty line.

**Table 3:** Changes in neighborhood-attributable variation in Prevention Quality Indicator visit likelihood by Census Block Group according to multilevel model results.

Model	ICC, % (95% CI)	Median OR
Model A: adjusted only for clustering by Census Block Group	5.92 (5.20, 6.73)	1.54
Model B: adjusted for patient characteristics and clustering by Census Block Group	4.97 (4.28, 5.80)	1.49
Model C: adjusted for patient characteristics, hospital distance, and clustering by Census Block Group	4.84 (4.16, 5.63)	1.48
Model D: adjusted for patient characteristics, hospital distance, Census Block Group -level socioeconomic indicators, and clustering by Census Block Group	4.11 (3.47, 4.87)	1.43

*ICC*, intraclass correlation coefficient; *CI*, confidence interval; *OR*, odds ratio.
