# Supplementary figures and images for: The Association of Demographic, Socioeconomic, and Geographic Factors with Potentially Preventable Emergency Department Utilization
Source: West J Emerg Med. 2021 Oct 27;22(6):1283–90. doi: 10.5811/westjem.2021.5.50233 (PMC8597685; doi:10.5811/westjem.2021.5.50233)

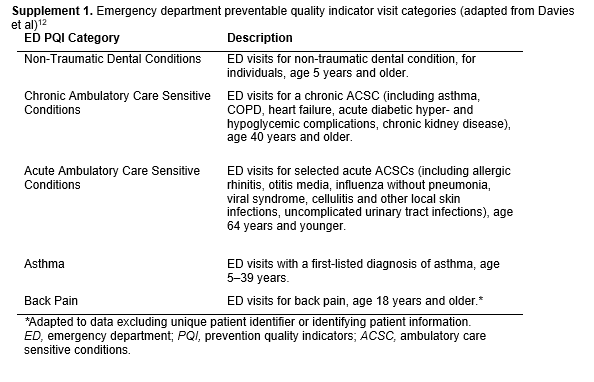

Supplement: Supplementary file 1 [file wjem-22-1283-s001.png]
